# 3-Hy­droxy-2-(4-meth­oxy­phen­yl)-4*H*-chromen-4-one

**DOI:** 10.1107/S160053681100167X

**Published:** 2011-01-22

**Authors:** Michał Wera, Ilia E. Serdiuk, Alexander D. Roshal, Jerzy Błażejowski

**Affiliations:** a Faculty of Chemistry, University of Gdańsk, J. Sobieskiego 18, 80-952 Gdańsk, Poland; b Institute of Chemistry, V.N. Karazin National University, Svobody 4, 61077 Kharkiv, Ukraine

## Abstract

In the title compound, C_16_H_12_O_4_, the benzene ring is twisted at an angle of 12.3 (1)° relative to the 4*H*-chromene skeleton, and an intramolecular O—H⋯O hydrogen bond occurs. The meth­oxy group is almost coplanar with the benzene ring [1.5 (1)°]. In the crystal, inversely oriented mol­ecules are arranged in double (*A*, *A*′) columns, along the *b* axis, and are linked by a network of inter­molecular O—H⋯O hydrogen bonds (between *A* and *A*′) and C—H⋯π contacts (within *A* or *A*′). The 4*H*-chromene cores are parallel within *A* or *A*′, but make a dihedral angle of 88.6 (1)° between *A* and *A*′.

## Related literature

For general features of flavonols (derivatives of 3-hy­droxy-2-phenyl-4*H*-chromen-4-one), see: Demchenko (2009 ▶); Klymchenko *et al.* (2003 ▶); Sengupta & Kasha (1979 ▶). For related structures, see: Etter *et al.* (1986 ▶); Waller *et al.* (2003 ▶); Wera *et al.* (2011 ▶). For inter­molecular inter­actions, see: Aakeröy *et al.* (1992 ▶); Takahashi *et al.* (2001 ▶). For the synthesis, see: Sobottka *et al.* (2000 ▶).
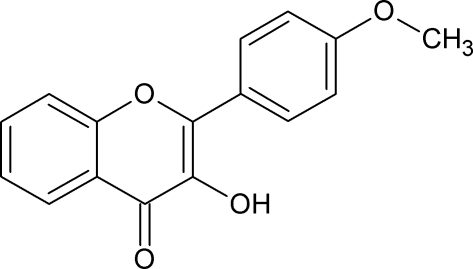


## Experimental

### 

#### Crystal data

C_16_H_12_O_4_
*M* *_r_* = 268.26
Monoclinic, 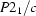

*a* = 11.2400 (5) Å
*b* = 4.9860 (2) Å
*c* = 21.9907 (9) Å
β = 95.116 (4)°
*V* = 1227.51 (9) Å^3^
*Z* = 4
Cu *K*α radiation
μ = 0.87 mm^−1^
*T* = 295 K
0.4 × 0.05 × 0.05 mm

#### Data collection

Oxford Diffraction Gemini R Ultra Ruby CCD diffractometer
Absorption correction: multi-scan (*CrysAlis RED*; Oxford Diffraction, 2008 ▶) *T*_min_ = 0.723, *T*_max_ = 0.888
7763 measured reflections
2209 independent reflections
1691 reflections with *I* > 2σ(*I*)
*R*_int_ = 0.031

#### Refinement

*R*[*F* ^2^ > 2σ(*F* ^2^)] = 0.040
*wR*(*F* ^2^) = 0.112
*S* = 1.04
2209 reflections
185 parameters
H atoms treated by a mixture of independent and constrained refinement
Δρ_max_ = 0.14 e Å^−3^
Δρ_min_ = −0.19 e Å^−3^

### 

Data collection: *CrysAlis CCD* (Oxford Diffraction, 2008 ▶); cell refinement: *CrysAlis RED* (Oxford Diffraction, 2008 ▶); data reduction: *CrysAlis RED*; program(s) used to solve structure: *SHELXS97* (Sheldrick, 2008 ▶); program(s) used to refine structure: *SHELXL97* (Sheldrick, 2008 ▶); molecular graphics: *ORTEP-3* (Farrugia, 1997 ▶); software used to prepare material for publication: *SHELXL97* and *PLATON* (Spek, 2009 ▶).

## Supplementary Material

Crystal structure: contains datablocks global, I. DOI: 10.1107/S160053681100167X/om2398sup1.cif

Structure factors: contains datablocks I. DOI: 10.1107/S160053681100167X/om2398Isup2.hkl

Additional supplementary materials:  crystallographic information; 3D view; checkCIF report

## Figures and Tables

**Table 1:** Hydrogen-bond geometry (Å, °) *Cg*1 is the centroid of the C13–C18 ring.

*D*—H⋯*A*	*D*—H	H⋯*A*	*D*⋯*A*	*D*—H⋯*A*
O11—H11⋯O12	0.91 (3)	2.17 (3)	2.672 (2)	114 (2)
O11—H11⋯O12^i^	0.91 (3)	1.92 (3)	2.748 (2)	149 (2)
C20—H20*B*⋯*Cg*1^ii^	0.96	2.87	3.710 (2)	147

Symmetry codes: (i) 
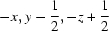
; (ii) 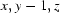.

**Fig. 1.**
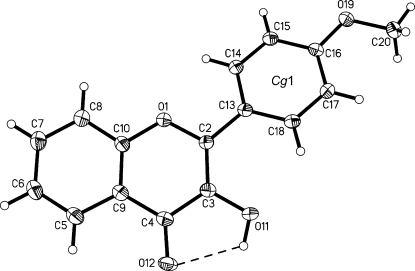
The molecular structure of the title compound showing the atom labeling scheme. Displacement ellipsoids are drawn at the 25% probability level, and H atoms are shown as small spheres of arbitrary radius. The O–H···O hydrogen bond is indicated by a dashed line.

**Fig. 2.**
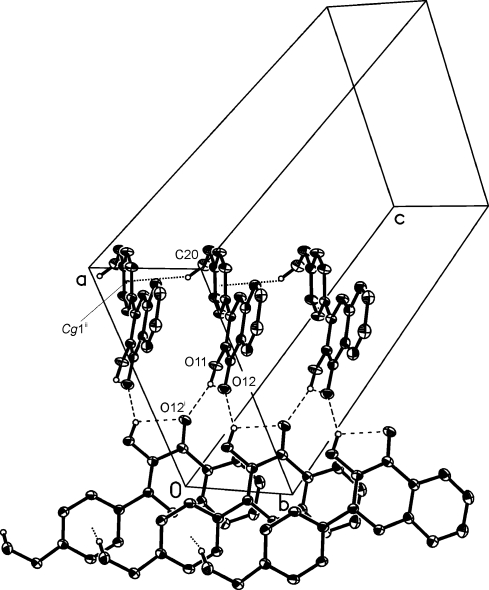
The arrangement of the molecules in the crystal structure. The O–H···O hydrogen bonds are represented by dashed lines, the C–H···π contacts by dotted lines. H atoms not involved in interactions have been omitted. [Symmetry codes: (i) –x, y – 1/2, –z + 1/2; (ii) x, y – 1, z.]

**Fig. 3.**
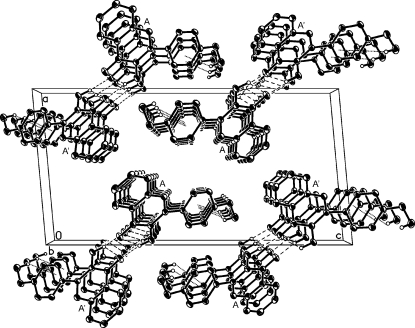
Columns in the crystal structure, viewed along the b axis. The O–H···O interactions are represented by dashed lines, the C–H···π contacts by dotted lines. H atoms not involved in interactions have been omitted. A and A' indicate the double columns.

## supplementary crystallographic information

### Comment

3-Hydroxy-2-phenyl-4*H*-chromen-4-one (flavonol) and its derivatives exhibit dual fluorescence in liquids arising from Excited State Intramolecular Proton Transfer (ESIPT) (Sengupta & Kasha, 1979). Both ESIPT and the fluorescence of flavonols depend substantially on the structure of the compounds (the angle between 4*H*-chromene and benzene moieties (Klymchenko *et al.*, 2003)) and the properties of the medium, which makes them convenient analytical probes in chemistry, biochemistry, biology and medicine (Demchenko, 2009). Here we present the crystal structure of a flavonol derivative – 3-hydroxy-2-(4-methoxyphenyl)-4*H*-chromen-4-one – a potential fluorescence sensor.

In the title compound (Fig. 1), the bond lengths and angles characterizing the geometry of the 4*H*-chromen-4-one moiety are similar to those in 2-phenyl-4*H*-chromen-4-one (flavone) (Waller *et al.*, 2003) and 3-hydroxy-2-phenyl-4*H*-chromen-4-one (flavonol) (Etter *et al.*, 1986). With respective average deviations from planarity of 0.0070 (2)° and 0.0055 (2)°, the 4*H*-chromene and benzene ring systems are oriented at a dihedral angle of 12.3 (1)° (in the case of flavonol this angle is equal to 5.5 (1)° (Etter *et al.*, 1986), while 3-hydroxy-2-(4-hydroxyphenyl)-4*H*-chromen-4-one – 20.7 (1)° (Wera *et al.*, 2011)). The methoxy group remains almost in the plane of the benzene ring: it is twisted relative to the benzene ring by an angle of only 1.5 (1)°.

In the crystal structure, the inversely oriented molecules are arranged in double (A,A') columns, along the *b* axis, and linked by a network of intermolecular O–H···O (Aakeröy *et al.*, 1992) hydrogen bonds (between A and A') and C–H···π (Takahashi *et al.*, 2001) contacts (within A or A') (Table 1, Figs. 2 and 3). The 4*H*-chromene cores are parallel within A or A', but lie at an angle of 88.6 (1)° between A and A'. The crystal lattice is stabilized by dispersive interactions between inversely oriented columns. The intramolecular O11–H11···O12 hydrogen bond (Table 1, Figs. 1–3) is believed to be involved in the ESIPT phenomenon, characteristic of flavonols (Sengupta & Kasha, 1979).

### Experimental

The title compound was synthesized as a result of the oxidative heterocyclization of 1-(2-hydroxyphenyl)-3-(4-methoxyphenyl)prop-2-en-1-one, synthesized first by the condensation of 1-(2-hydroxyphenyl)ethanone with 4-methoxybenzaldehyde in ethanol/50% aqueous NaOH (1/1 *v*/*v*), in alkaline ethanol/H_2_O_2_ (Sobottka *et al.*, 2000). The filtered product was purified chromatographically (Silica Gel, chloroform/ethanol, 20/1 *v*/*v*) and colorless crystals suitable for X-ray investigations were grown from chloroform (m.p. = 510 – 511 K).

### Refinement

H atoms of C–H bonds were positioned geometrically, with C–H = 0.93Å and 0.96Å for the aromatic and methyl H atoms, respectively, and constrained to ride on their parent atoms with *U*_iso_(H) = *xU*_eq_(C) where *x* = 1.2 for the aromatic H and 1.5 for methyl H atoms. H atoms involved in O–H···O hydrogen bonds were located on a difference Fourier map and refined isotropically with *U*_iso_(H) = 1.5*U*_eq_(O).

### Crystal data

**Table:**

C_16_H_12_O_4_	*F*(000) = 560
*M**_r_* = 268.26	*D*_x_ = 1.452 Mg m^−^^3^
Monoclinic, *P*2_1_/*c*	Cu *K*α radiation, λ = 1.54184 Å
Hall symbol: -P 2ybc	Cell parameters from 2209 reflections
*a* = 11.2400 (5) Å	θ = 4.0–68.3°
*b* = 4.9860 (2) Å	µ = 0.87 mm^−^^1^
*c* = 21.9907 (9) Å	*T* = 295 K
β = 95.116 (4)°	Needle, colorless
*V* = 1227.51 (9) Å^3^	0.4 × 0.05 × 0.05 mm
*Z* = 4	

### Data collection

**Table:**

Oxford Diffraction Gemini R Ultra Ruby CCD diffractometer	2209 independent reflections
Radiation source: Ultra (Cu) X-ray Source'	1691 reflections with *I* > 2σ(*I*)
mirror	*R*_int_ = 0.031
Detector resolution: 10.4002 pixels mm^-1^	θ_max_ = 68.3°, θ_min_ = 4.0°
ω scans	*h* = −13→13
Absorption correction: multi-scan (*CrysAlis RED*; Oxford Diffraction, 2008)	*k* = −5→5
*T*_min_ = 0.723, *T*_max_ = 0.888	*l* = −25→26
7763 measured reflections	

### Refinement

**Table:**

Refinement on *F*^2^	Primary atom site location: structure-invariant direct methods
Least-squares matrix: full	Secondary atom site location: difference Fourier map
*R*[*F*^2^ > 2σ(*F*^2^)] = 0.040	Hydrogen site location: inferred from neighbouring sites
*wR*(*F*^2^) = 0.112	H atoms treated by a mixture of independent and constrained refinement
*S* = 1.04	*w* = 1/[σ^2^(*F*_o_^2^) + (0.056*P*)^2^ + 0.2165*P*] where *P* = (*F*_o_^2^ + 2*F*_c_^2^)/3
2209 reflections	(Δ/σ)_max_ < 0.001
185 parameters	Δρ_max_ = 0.14 e Å^−^^3^
0 restraints	Δρ_min_ = −0.19 e Å^−^^3^

### Special details

**Table:**

Geometry. All e.s.d.'s (except the e.s.d. in the dihedral angle between two l.s. planes) are estimated using the full covariance matrix. The cell e.s.d.'s are taken into account individually in the estimation of e.s.d.'s in distances, angles and torsion angles; correlations between e.s.d.'s in cell parameters are only used when they are defined by crystal symmetry. An approximate (isotropic) treatment of cell e.s.d.'s is used for estimating e.s.d.'s involving l.s. planes.
Refinement. Refinement of *F*^2^ against ALL reflections. The weighted *R*-factor *wR* and goodness of fit *S* are based on *F*^2^, conventional *R*-factors *R* are based on *F*, with *F* set to zero for negative *F*^2^. The threshold expression of *F*^2^ > σ(*F*^2^) is used only for calculating *R*-factors(gt) *etc*. and is not relevant to the choice of reflections for refinement. *R*-factors based on *F*^2^ are statistically about twice as large as those based on *F*, and *R*- factors based on ALL data will be even larger.

### Fractional atomic coordinates and isotropic or equivalent isotropic displacement parameters (Å^2^)

**Table:**

	*x*	*y*	*z*	*U*_iso_*/*U*_eq_	
O1	0.31792 (10)	0.8649 (2)	0.40435 (5)	0.0418 (3)	
C2	0.22283 (14)	0.6906 (3)	0.40261 (7)	0.0360 (4)	
C3	0.14034 (15)	0.6880 (3)	0.35333 (7)	0.0382 (4)	
C4	0.15007 (15)	0.8610 (3)	0.30130 (7)	0.0387 (4)	
C5	0.27235 (18)	1.2220 (4)	0.25831 (8)	0.0481 (5)	
H5	0.2193	1.2302	0.2234	0.058*	
C6	0.37020 (19)	1.3850 (4)	0.26374 (9)	0.0526 (5)	
H6	0.3833	1.5046	0.2326	0.063*	
C7	0.45087 (18)	1.3732 (4)	0.31592 (9)	0.0510 (5)	
H7	0.5176	1.4843	0.3191	0.061*	
C8	0.43220 (16)	1.1982 (4)	0.36261 (8)	0.0459 (4)	
H8	0.4857	1.1900	0.3973	0.055*	
C9	0.25145 (15)	1.0416 (3)	0.30532 (7)	0.0385 (4)	
C10	0.33197 (15)	1.0345 (3)	0.35681 (7)	0.0391 (4)	
O11	0.04691 (11)	0.5154 (3)	0.35012 (5)	0.0495 (3)	
H11	0.012 (2)	0.521 (5)	0.3111 (11)	0.074*	
O12	0.07565 (12)	0.8432 (3)	0.25643 (5)	0.0523 (4)	
C13	0.22946 (15)	0.5200 (3)	0.45717 (7)	0.0366 (4)	
C14	0.33350 (16)	0.5131 (4)	0.49652 (8)	0.0458 (4)	
H14	0.3973	0.6231	0.4888	0.055*	
C15	0.34395 (16)	0.3468 (4)	0.54671 (8)	0.0485 (5)	
H15	0.4151	0.3426	0.5718	0.058*	
C16	0.24935 (16)	0.1858 (4)	0.56016 (7)	0.0411 (4)	
C17	0.14504 (16)	0.1926 (4)	0.52235 (8)	0.0494 (5)	
H17	0.0806	0.0867	0.5311	0.059*	
C18	0.13582 (16)	0.3565 (4)	0.47141 (8)	0.0479 (5)	
H18	0.0651	0.3573	0.4460	0.057*	
O19	0.26808 (12)	0.0309 (3)	0.61117 (5)	0.0532 (4)	
C20	0.17391 (19)	−0.1423 (4)	0.62537 (9)	0.0568 (5)	
H20A	0.1990	−0.2448	0.6612	0.085*	
H20B	0.1541	−0.2616	0.5917	0.085*	
H20C	0.1051	−0.0374	0.6328	0.085*	

### Atomic displacement parameters (Å^2^)

**Table:**

	*U*^11^	*U*^22^	*U*^33^	*U*^12^	*U*^13^	*U*^23^
O1	0.0424 (6)	0.0461 (7)	0.0357 (6)	−0.0079 (5)	−0.0038 (5)	0.0054 (5)
C2	0.0354 (8)	0.0395 (9)	0.0327 (8)	−0.0025 (7)	0.0005 (6)	−0.0004 (7)
C3	0.0389 (9)	0.0418 (9)	0.0333 (8)	−0.0001 (7)	−0.0004 (7)	−0.0013 (7)
C4	0.0432 (9)	0.0391 (9)	0.0332 (8)	0.0070 (7)	−0.0005 (7)	−0.0010 (7)
C5	0.0617 (12)	0.0450 (10)	0.0375 (9)	0.0048 (9)	0.0042 (8)	0.0035 (7)
C6	0.0662 (12)	0.0469 (11)	0.0465 (10)	0.0011 (9)	0.0142 (9)	0.0090 (8)
C7	0.0536 (11)	0.0467 (10)	0.0542 (11)	−0.0053 (9)	0.0139 (9)	0.0017 (8)
C8	0.0460 (10)	0.0448 (10)	0.0467 (10)	−0.0030 (8)	0.0037 (8)	0.0009 (8)
C9	0.0449 (9)	0.0361 (9)	0.0347 (8)	0.0063 (7)	0.0046 (7)	−0.0004 (7)
C10	0.0456 (9)	0.0370 (9)	0.0352 (8)	0.0015 (7)	0.0059 (7)	0.0020 (7)
O11	0.0479 (7)	0.0638 (8)	0.0342 (6)	−0.0158 (6)	−0.0108 (5)	0.0056 (6)
O12	0.0593 (8)	0.0566 (8)	0.0376 (7)	0.0003 (6)	−0.0140 (6)	0.0054 (6)
C13	0.0378 (8)	0.0407 (9)	0.0307 (8)	−0.0016 (7)	0.0001 (6)	−0.0009 (7)
C14	0.0400 (9)	0.0572 (11)	0.0391 (9)	−0.0129 (8)	−0.0032 (7)	0.0069 (8)
C15	0.0401 (9)	0.0629 (12)	0.0401 (9)	−0.0094 (9)	−0.0101 (7)	0.0102 (8)
C16	0.0475 (10)	0.0455 (9)	0.0296 (8)	−0.0040 (8)	−0.0013 (7)	0.0019 (7)
C17	0.0444 (10)	0.0587 (11)	0.0438 (10)	−0.0160 (9)	−0.0037 (8)	0.0076 (8)
C18	0.0387 (9)	0.0626 (12)	0.0401 (9)	−0.0094 (9)	−0.0093 (7)	0.0091 (8)
O19	0.0547 (8)	0.0630 (8)	0.0397 (7)	−0.0141 (6)	−0.0076 (6)	0.0156 (6)
C20	0.0661 (13)	0.0593 (12)	0.0447 (10)	−0.0167 (10)	0.0025 (9)	0.0116 (9)

### Geometric parameters (Å, °)

**Table:**

O1—C10	1.3645 (19)	C9—C10	1.386 (2)
O1—C2	1.3755 (19)	O11—H11	0.91 (3)
C2—C3	1.362 (2)	C13—C18	1.389 (2)
C2—C13	1.467 (2)	C13—C14	1.392 (2)
C3—O11	1.355 (2)	C14—C15	1.377 (2)
C3—C4	1.445 (2)	C14—H14	0.9300
C4—O12	1.238 (2)	C15—C16	1.385 (2)
C4—C9	1.449 (2)	C15—H15	0.9300
C5—C6	1.364 (3)	C16—O19	1.363 (2)
C5—C9	1.406 (2)	C16—C17	1.376 (2)
C5—H5	0.9300	C17—C18	1.383 (2)
C6—C7	1.399 (3)	C17—H17	0.9300
C6—H6	0.9300	C18—H18	0.9300
C7—C8	1.378 (3)	O19—C20	1.422 (2)
C7—H7	0.9300	C20—H20A	0.9600
C8—C10	1.388 (2)	C20—H20B	0.9600
C8—H8	0.9300	C20—H20C	0.9600
			
C10—O1—C2	120.85 (13)	C9—C10—C8	121.70 (16)
C3—C2—O1	120.02 (14)	C3—O11—H11	107.3 (15)
C3—C2—C13	128.80 (15)	C18—C13—C14	117.19 (15)
O1—C2—C13	111.15 (13)	C18—C13—C2	122.71 (14)
O11—C3—C2	121.13 (15)	C14—C13—C2	120.09 (15)
O11—C3—C4	116.80 (14)	C15—C14—C13	121.33 (16)
C2—C3—C4	122.03 (15)	C15—C14—H14	119.3
O12—C4—C3	119.67 (16)	C13—C14—H14	119.3
O12—C4—C9	124.37 (15)	C14—C15—C16	120.53 (16)
C3—C4—C9	115.93 (14)	C14—C15—H15	119.7
C6—C5—C9	120.20 (17)	C16—C15—H15	119.7
C6—C5—H5	119.9	O19—C16—C17	124.91 (16)
C9—C5—H5	119.9	O19—C16—C15	116.04 (15)
C5—C6—C7	120.35 (17)	C17—C16—C15	119.04 (16)
C5—C6—H6	119.8	C16—C17—C18	120.16 (17)
C7—C6—H6	119.8	C16—C17—H17	119.9
C8—C7—C6	120.48 (18)	C18—C17—H17	119.9
C8—C7—H7	119.8	C17—C18—C13	121.73 (16)
C6—C7—H7	119.8	C17—C18—H18	119.1
C7—C8—C10	118.72 (18)	C13—C18—H18	119.1
C7—C8—H8	120.6	C16—O19—C20	117.48 (14)
C10—C8—H8	120.6	O19—C20—H20A	109.5
C10—C9—C5	118.54 (16)	O19—C20—H20B	109.5
C10—C9—C4	119.12 (15)	H20A—C20—H20B	109.5
C5—C9—C4	122.33 (16)	O19—C20—H20C	109.5
O1—C10—C9	122.03 (15)	H20A—C20—H20C	109.5
O1—C10—C8	116.27 (15)	H20B—C20—H20C	109.5
			
C10—O1—C2—C3	−0.8 (2)	C4—C9—C10—O1	−0.9 (2)
C10—O1—C2—C13	177.25 (14)	C5—C9—C10—C8	−0.6 (3)
O1—C2—C3—O11	178.96 (15)	C4—C9—C10—C8	178.47 (16)
C13—C2—C3—O11	1.3 (3)	C7—C8—C10—O1	179.94 (15)
O1—C2—C3—C4	1.2 (2)	C7—C8—C10—C9	0.5 (3)
C13—C2—C3—C4	−176.51 (16)	C3—C2—C13—C18	−12.4 (3)
O11—C3—C4—O12	−1.0 (2)	O1—C2—C13—C18	169.72 (15)
C2—C3—C4—O12	176.88 (16)	C3—C2—C13—C14	166.24 (18)
O11—C3—C4—C9	−179.22 (14)	O1—C2—C13—C14	−11.6 (2)
C2—C3—C4—C9	−1.4 (2)	C18—C13—C14—C15	1.4 (3)
C9—C5—C6—C7	0.3 (3)	C2—C13—C14—C15	−177.35 (17)
C5—C6—C7—C8	−0.4 (3)	C13—C14—C15—C16	−1.6 (3)
C6—C7—C8—C10	0.0 (3)	C14—C15—C16—O19	−179.23 (17)
C6—C5—C9—C10	0.1 (3)	C14—C15—C16—C17	0.6 (3)
C6—C5—C9—C4	−178.88 (16)	O19—C16—C17—C18	−179.65 (17)
O12—C4—C9—C10	−176.95 (16)	C15—C16—C17—C18	0.6 (3)
C3—C4—C9—C10	1.2 (2)	C16—C17—C18—C13	−0.7 (3)
O12—C4—C9—C5	2.0 (3)	C14—C13—C18—C17	−0.2 (3)
C3—C4—C9—C5	−179.82 (16)	C2—C13—C18—C17	178.47 (17)
C2—O1—C10—C9	0.7 (2)	C17—C16—O19—C20	1.9 (3)
C2—O1—C10—C8	−178.72 (14)	C15—C16—O19—C20	−178.31 (16)
C5—C9—C10—O1	−179.96 (15)		

### Hydrogen-bond geometry (Å, °)

**Table:**

*Cg*1 is the centroid of the C13–C18 ring.

**Table:**

*D*—H···*A*	*D*—H	H···*A*	*D*···*A*	*D*—H···*A*
O11—H11···O12	0.91 (3)	2.17 (3)	2.672 (2)	114 (2)
O11—H11···O12^i^	0.91 (3)	1.92 (3)	2.748 (2)	149 (2)
C20—H20B···Cg1^ii^	0.96	2.87	3.710 (2)	147

Symmetry codes:  (i) −*x*, *y*−1/2, −*z*+1/2; (ii) *x*, *y*−1, *z*.
